# The Thermal Adaptability of *Sclerodermus guani* Xiao *et* Wu (Hymenoptera: Bethylidae), an Important Parasitoid of Long-Horned Beetles in China

**DOI:** 10.3390/biology14091234

**Published:** 2025-09-10

**Authors:** Lina Wang, Yuhua Situ, Jie Zhang, Kui Kang, Zhongjiu Xiao, Shaobo Wang, Ke Wei, Yanlong Tang

**Affiliations:** 1 College of Biology and Agriculture, Zunyi Normal University, Zunyi 563002, China; 2 Centre of Earth Observation Science, University of Manitoba, Winnipeg, MB R3T 2N2, Canada; 3 Key Laboratory of Forest Protection of National Forestry and Grassland Administration, Ecology and Nature Conservation Institute, Chinese Academy of Forestry, Beijing 100091, China

**Keywords:** *Sclerodermus guani* Xiao *et* Wu, temperature, development, reproduction

## Abstract

*Sclerodermus guani* is the most extensively studied and widely used parasitoid wasp for controlling forest trunk-boring pests in China, with temperature being a key environmental factor affecting its development and reproduction. Against the backdrop of global climate change, the frequency of heat stress on parasitoid wasps is increasing. Therefore, studying their adaptability to heat stress is of great significance for breeding heat-tolerant strains. The findings of such research may help improve efficiency in rearing this wasp and provide guidance for its use in pest control. This study investigated the thermal adaptability of the wasp at temperatures ranging from 21 to 33 °C. The results showed that temperature significantly affected its parasitism capacity and behavior. As temperature increased, female wasps searched for and stung hosts more actively, and their pre-oviposition period decreased, as well as their developmental duration. The reproductive fitness of the wasp also varied significantly with temperature, with the most favorable temperatures being 27 °C and 30 °C. Both low and high temperatures were detrimental to its reproduction, and the difference in the number of female offspring was significant. The experimental results indicate that *S. guani* has higher reproductive fitness at 27–30 °C. Therefore, it is recommended that this parasitoid wasp be reared indoors at 27–30 °C and released for pest control in forests when the temperature is above 24 °C.

## Simple Summary

*Sclerodermus guani* Xiao *et* Wu is an important parasitoid of long-horned beetles in China and has been widely used to control forest trunk-boring pests for over 40 years. This study aimed to determine the adaptive characteristics of this parasitoid wasp in response to temperature variations. Between 21 and 33 °C, the parasitoid behavior and parasitism capacity of the wasp varied significantly. Higher temperatures resulted in more-active females, a shorter supplementary feeding period, and a reduced developmental duration in offspring. At the highest temperature (33 °C), offspring development was most rapid, but the number of offspring was the lowest. Conversely, at the lowest temperature (21 °C), offspring development was the slowest, the number of offspring was similarly low, and parasitism and offspring emergence rates were at their minimum. These results indicate that *S. guani* exhibits higher developmental fitness between 24 and 30 °C, while its reproductive fitness is the highest between 27 and 30 °C. Therefore, it is recommended that this parasitoid wasp be reared indoors at 27 and 30 °C and released for pest control in forests when the temperature is above 24 °C.

## 1. Introduction

The impacts of wood-boring beetles on forests and crops necessitate more-efficient parasitoids under climate change scenarios. *Sclerodermus guani* was discovered in the 1970s in China’s Shandong and Guangdong provinces [1,2], and it was subsequently found to parasitize the larvae and pupae of small- to medium-sized long-horned beetles, such as *Saperda populnea* and *S. sinoauster* [3]. It can also effectively parasitize the first- to third-instar larvae of larger long-horned beetles such as *Monochamus alternatus* and *Anoplophora glabripennis* in China [4]. Consequently, *S. guani* has gradually become the most intensively studied and widely used parasitoid wasp for controlling forest trunk-boring pests in China [4]. In this country, the life stages at which these beetle pests are most susceptible to parasitism by *S. guani* largely occur in July and August. For example, in this period, *S. bifasciatus* and *S. sinoauster* are in their mature larval stage, and while *M. alternatus* and *A. glabripennis* are mostly in their first to third larval instars [4]. July and August are also the hottest months in the Northern Hemisphere. In recent years, as global warming has made high summer temperatures the norm, studying the thermal adaptation characteristics of this parasitoid wasp, which inhabits the warm, temperate, monsoon climate zone of the Northern Hemisphere, has become of great significance for its mass rearing and application.

Ambient temperature is a primary determinant of an insect’s performance and ecological success [5,6]. Changes in temperature can affect an organism’s specific biological processes, including behavior, growth and developmental rates, fecundity, and physiological metabolism [7,8,9]. Generally, the developmental duration of parasitoid wasps shortens as temperature increases, as seen in *Tetrastichus planipennisi* [10], *Nasonia vitripennis* [11], *Cyanopterus ninghais* [12], *S. alternatusi* [13], and *S. pupariae* [14]. However, at higher temperatures, the reproduction of parasitoid wasps is limited. For example, while no parasitoid eggs of *T. planipennisi* hatch at 35 °C [10], the eggs of *N. vitripennis* also fail to hatch at 34 °C [11], and the number of offspring of *S. alternatusi* and *S. pupariae* decreases significantly at 33 °C [13,14]. Temperature also has a significant impact on the parasitoid behavior and activity of wasps. The parasitism rates of *S. alternatusi* and *S. pupariae* decline markedly at 33 °C [13,14], whereas the parasitism rate of *C. ninghais* is the highest at 29 °C, with both low and high temperatures adversely affecting its parasitoid efficiency [12]. Understanding how *S. guani* tolerates high temperatures is essential for its use in biocontrol under climate change scenarios, yet no data exist for temperatures > 30 °C. In addition, under rising temperature conditions, the developmental duration of the host is shortened, with environmental temperature variations reducing the host presence window, thus shortening the period during which hosts at susceptible stages are available [15].

Wasps of the genus *Sclerodermus* are ectoparasitoids. Before oviposition, they undergo a series of processes including searching for a host, stinging it and injecting it with venom to paralyze it, cleaning debris from the surface of the host’s body, and feeding on the host’s hemolymph for nutritional supplementation [16,17]. Temperature significantly affects the vitality of these wasps. For *S. alternatusi*, the time from introduction to host paralysis is 6.7 days at 21 °C but only 1.5 days at 30 °C; the subsequent period until oviposition is 9.3 days long at 21 °C but only 4.4 days long at 33 °C [13]. For *S. pupariae*, the time from introduction to oviposition is about 12 days at 21 °C, shortening to less than 3 days at 33 °C [14]. In the case of *S. sichuanensis*, the supplementary feeding period is about 20 days in April (spring) but only about 6 days in July (summer) [18]. However, the relationship between the supplementary feeding period of *S. guani* and temperature has not been reported. Although a few studies have described the effects of temperature on the development and reproduction of *S. guani*, none have included data collected at high temperatures above 30 °C [3,19]. Against the backdrop of global warming, insects are experiencing heat stress more frequently, and parasitoid wasps are no exception. The response and adaptability of parasitoid wasps to high temperatures are therefore current research hotspots. Here, we examined the effects of high temperatures (>30 °C) on the development, reproduction, and parasitism efficiency of *S. guani*, with the aim of elucidating its thermal tolerance and potential in biocontrol under climate change.

## 2. Materials and Methods

### 2.1. Insects

*Sclerodermus guani* was collected in 2022 at the Xishan Experimental Forest Farm in Beijing (116.19° E, 39.99° N) and reared on larvae of *Thyestilla gebleri*, which acted as hosts, in the laboratory. The rearing conditions were a temperature of 25 °C, 60–70% relative humidity (RH), a photoperiod of 8 h L:16 h D, and a light intensity of 3000 lx. Healthy and active 3-day-old female wasps that had been fully mated were selected for this study. The host insects used were last-instar larvae of *Thyestilla gebleri* weighing between 200.0 and 220.0 mg, with thirty replicates for each treatment collected from the roots of infested *Abutilon theophrasti* Medicus from Dagang District (38°56′ N, 117°29′ E), Tianjin City, China [20,21].

### 2.2. Inoculation of Sclerodermus guani

Individual hosts were placed in glass culture tubes (1.0 cm in diameter, 5.0 cm in length). Female wasps of similar size, with a body length of about 3.5 mm, were selected and introduced individually into a tube containing one *T. gebleri* larva. The tube openings were then sealed with cotton wool plugs, and water/sugar was not provided. The rearing temperatures were set to 21 °C, 24 °C, 27 °C, 30 °C, and 33 °C, with 30 tubes prepared for each treatment. Across temperature conditions, the humidity and photoperiod were kept constant (RH = 60–70%; photoperiod of 8 h L:16 h D; light intensity of 3000 lx).

### 2.3. Observation of the Development and Reproductive Fitness of Sclerodermus guani Under Different Rearing Temperatures

The parasitism capacity of the mother wasp on the host was recorded, including the parasitism rate and the offspring emergence rate. The parasitism rate was defined as the proportion of parasitized hosts to the total number of hosts provided. The offspring emergence rate was defined as the proportion of hosts from which offspring wasps emerged to the total number of hosts provided. The pre-oviposition period of the mother wasp, and the developmental duration and number of offspring wasps were also recorded. The pre-oviposition period is the interval from the introduction of the wasp to the observation of the first laid egg with a Motic stereoscope. The developmental duration of the offspring wasps included the egg, larval, pupal, and egg-to-adult durations. The egg duration is the interval from the laying of the first egg to the hatching of the first larva. The larval duration is the interval from the hatching of the first larva to the appearance of the first cocoon. The pupal duration is the interval from the appearance of the first cocoon to the emergence of the first adult wasp. Finally, the egg-to-adult duration is the time from the laying of the first egg to the emergence of the first adult wasp. Duration was measured in days. For each culture tube, the developmental duration of the offspring wasps was recorded once to represent the developmental progress of the offspring cohort, including the times at which the first egg was laid by the female, the first egg hatched, a first-instar larva cocooned, and the first adult emerged. After the complete emergence of the offspring wasps, the number of female and male wasps from each host and the sex ratio were calculated. The sex ratio is defined as the proportion of female wasps in all offspring of a single host [21,22]. All the above measurements were made once daily.

### 2.4. Developmental Threshold Temperature and Effective Accumulated Temperature for Each Life Stage of Sclerodermus guani

Based on the linear relationship between temperature and developmental rate, the effective accumulated temperature (*K*) and the developmental threshold temperature (*C*) required for *S. guani* to complete a specific life stage were calculated. The developmental rate (*V*) at different temperatures for each life stage was calculated using the following Equation:

(1) $$V=\frac{1}{N}\;(d^{-1})\;$$

where *V* is the daily developmental rate, and *N* is the number of days required for completion of each life stage of *S. guani*, obtained from the experiment in Section 2.3. A linear regression Equation,

(2) T=a+b×V 

was fitted with the experimental temperature (*T*) as the y-axis and the developmental rate (*V*) as the x-axis. Following standard linear methods [3], the x-intercept of the regression corresponds to the developmental threshold temperature (*C*), and the inverse of the slope represents the effective accumulated temperature (*K*).

### 2.5. Data Analysis and Statistics

Fisher’s exact test was used to examine the differences in the parasitism capacity of *S. guani* under different temperatures. For the pre-oviposition period, developmental duration, number of offspring, and sex ratio, data were first assessed for normality using the Shapiro–Wilk test and for homogeneity of variance using Levene’s test. Female sex ratio data were arcsine-transformed to meet these assumptions. One-way ANOVA was then performed to test for significant differences among treatments, followed by Fisher’s least significant difference (LSD) test for multiple comparisons. The linear regression equation was evaluated using an *F*-test. All statistical analyses were performed using SPSS 22.0.

## 3. Results

### 3.1. Effect of Temperature on the Parasitism Capacity of Sclerodermus guani

The parasitism capacity of *S. guani* was significantly influenced by temperature (Table 1). Across the five temperature gradients tested (21 °C to 33 °C), both the parasitism rate (*χ*^2^ = 11.606; *df* = 4; *p* = 0.021) and the F1 emergence rate (*χ*^2^ = 29.667; *df* = 4; *p <* 0.001) were significantly different. At the lowest temperature of 21 °C, the parasitism and emergence rates were at their minimum, at 66.67% and 46.67%, respectively. Both rates peaked at 96.67% at 24 °C. Subsequently, as temperatures increased further, both the parasitism and emergence rates exhibited a declining trend, with 90.00% at 27 °C, 86.67% at 30 °C, and a further decrease to 83.33% at 33 °C.

### 3.2. The Effect of Temperature on the Parasitoid Behavior of Sclerodermus guani

**Figure 1:**
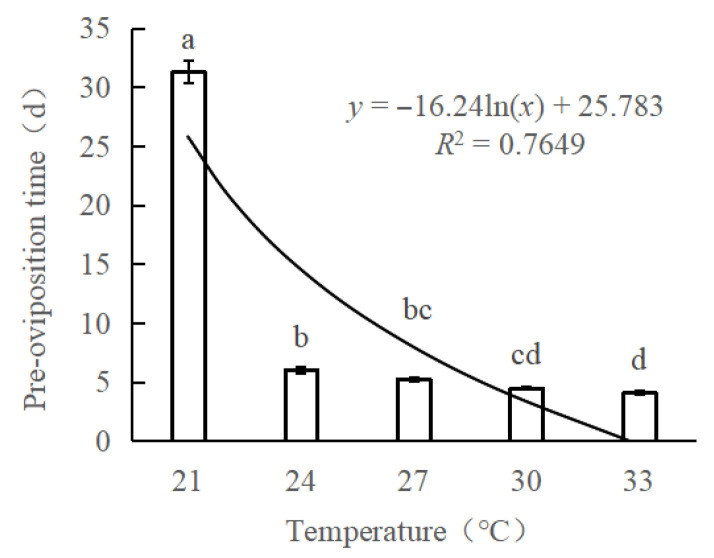
The pre-oviposition time of *Sclerodermus guani* females at different temperatures. Data are means ± SE of thirty replicates. Different letters above the columns indicate significant differences among the groups according to ANOVA performed with the LSD test (*p* < 0.05).

The results demonstrated that temperature significantly affects the parasitoid behavior of *S. guani*. Specifically, higher temperatures increased the activity level of the parasitoid wasps, leading to more-proactive host-seeking and stinging behaviors. Consequently, the pre-oviposition period was significantly shortened (*F* = 795.822; *df* = 4, 126; *p* < 0.001) (Figure 1). A significant negative correlation was observed between the pre-oviposition period and temperature (*y* = −16.24ln(*x*) + 25.783). At 21 °C, the pre-oviposition period was longest, averaging 31.3 days. This duration shortened dramatically to an average of 6.0 days at 24° C and continued to decrease to 5.3 and 4.5 days at 27 °C and 30 °C, respectively. The shortest pre-oviposition period was recorded at 33 °C, at which the wasps required only 4.1 days to complete host paralysis and nutritional feeding before commencing oviposition (Table S1).

### 3.3. Developmental Adaptability of Sclerodermus guani to Different Temperatures

**Figure 2:**
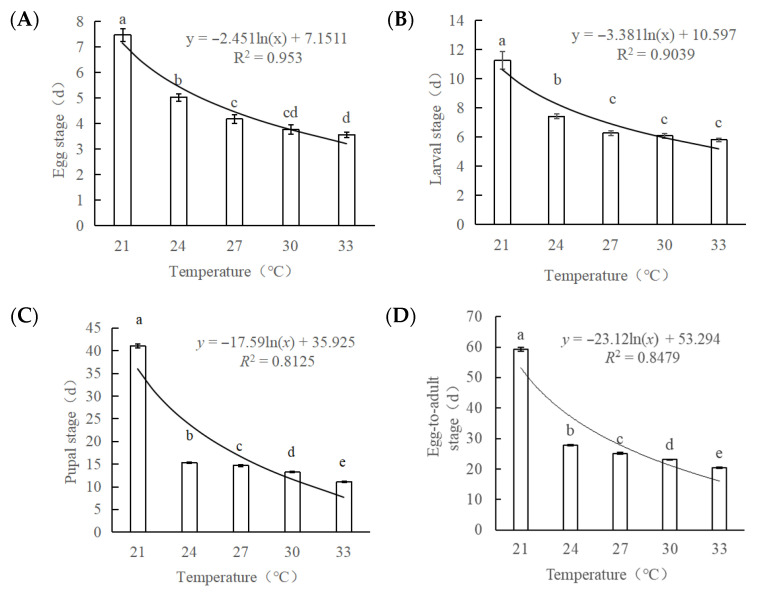
The developmental stages of *Sclerodermus guani* at different temperatures. (**A**): egg stage; (**B**): larval stage; (**C**): pupal stage; (**D**): egg-to-adult stage. Data are means ± SE of thirty replicates. Different letters above the columns indicate significant differences among the groups according to ANOVA performed with the LSD test (*p* < 0.05).

*Sclerodermus guani* exhibited distinct developmental adaptations to varying temperatures. The durations of the egg stage (*F* = 20.769; *df* = 2, 82; *p* < 0.001), larval stage (*F* = 23.239; *df* = 2, 78; *p* < 0.001), pupal stage (*F* = 74.504; *df* = 2, 77; *p* < 0.001), and the complete egg-to-adult stage (*F* = 151.390; *df* = 2, 77; *p* < 0.001) all decreased significantly with rising temperatures (Figure 2). The longest developmental periods were recorded at 21 °C, with average egg, larval, pupal, and egg-to-adult durations of 7.5, 11.3, 41.1, and 59.3 days, respectively. At 24 °C, these durations shortened significantly to 6.1, 6.4, 15.3, and 27.8 days, respectively. The shortest developmental times occurred at 33 °C, with durations of 4.6, 4.8, 11.1, and 20.4 days for the respective stages. Compared to the durations at 21 °C, these represent reductions of 2.9, 6.5, 30.0, and 38.9 days. The durations of the egg, larval, pupal, and egg-to-adult stages were all negatively correlated with temperature, as described by the following respective equations: *y* = −2.451ln(*x*) + 7.1511; *y* = −3.381ln(*x*) + 10.597; *y* = −17.59ln(*x*) + 35.925; and *y* = −23.12ln(*x*) + 53.294 (Table S1). 

### 3.4. Reproductive Fitness of Sclerodermus guani at Different Temperatures

**Figure 3:**
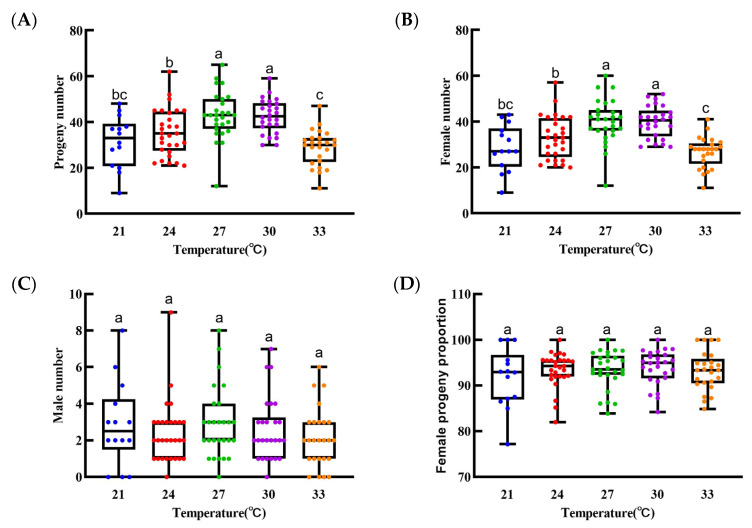
The brood size and female ratio of *Sclerodermus guani* progeny. (**A**): Progeny number; (**B**): Female number; (**C**): male number; (**D**): female ratio. Data are means ± SE of thirty replicates. Different letters above the bars indicate significant differences among the groups according to ANOVA performed with the LSD test (*p* < 0.05).

*Sclerodermus guani* exhibited significant differences in reproductive fitness across various temperatures. The optimal temperatures for reproduction were 27 °C and 30 °C, which yielded the most total progeny, averaging 43.0 and 42.5 individuals/tube, respectively (Figure 3). Conversely, both lower and higher temperatures were detrimental to reproduction. At 21 °C, the total offspring count was 31.4 per tube, while at 33 °C, it was at its lowest, at only 28.8 per tube—a significant reduction of 13.7 individuals from the peak value (*F* = 11.154; *df* = 4, 120; *p* < 0.001). The number of male offspring did not differ significantly across temperatures (*F* = 0.72; *df* = 4, 120; *p* = 0.580), with averages ranging from 2.2 to 2.9 individuals/tube. In contrast, the number of female offspring varied significantly (*F* = 12.598; *df* = 4, 120; *p* < 0.001). The most female progeny were observed at 27 °C (mean of 40.1 per tube), followed closely by 30 °C (mean of 39.9 per tube). However, the number dropped sharply to 26.6 per tube at 33 °C, 13.5 individuals fewer than the maximum (Table S2). Despite these variations, the sex ratio was consistently female-biased, with the proportion of females exceeding 90% and showing no significant differences across all treatments (*F* = 0.615; *df* = 4, 120; *p* = 0.652) (Table S3). This confirms that *Sclerodermus guani* is a species with a typically female-biased sex ratio. 

### 3.5. Developmental Thresholds and Developmental Threshold Temperatures for Different Life Stages of Sclerodermus guani

The developmental rates of *S. guani* at various temperatures are presented in Table 2. The rates for the egg, larval, and pupal stages ranged from 0.1368 to 0.2867, 0.0932 to 0.1741, and 0.0244 to 0.0908, respectively. Based on these data, the lower developmental threshold temperature (T0) for the egg stage was estimated to be 10.19 °C, with an effective accumulated temperature (K) of 72.57 degree-days. For the larval stage, the developmental threshold was 7.73 °C and the effective accumulated temperature was 131.21 degree-days. Finally, for the pupal stage, the developmental threshold was 15.57 °C and the effective accumulated temperature was 176.02 degree-days (Table 3).

## 4. Discussion

Currently, all reported parasitoids of the genus *Sclerodermus* are synovigenic. Females rely on host-feeding to supplement their nutrition for the development of their ovaries and oocytes, whereas males can mate without nutrient supplementation [1,18,23,24]. After female wasps supplement their nutrition, their abdominal intersegmental membranes stretch, and their body length increases by more than one-third [25]. Studies have shown that the duration of the nutrient supplementation period for females of the genus *Sclerodermus* is closely related to temperature. For *S. alternatusi*, this period can be as long as 15 days at 21 °C, but it shortens rapidly when the temperature rises above 24 °C [13]. For *S. guani*, the pre-oviposition period is as long as 31.3 days at 21 °C, shortening significantly at 24 °C, and at 33 °C, the processes of stinging and paralyzing the host and nutrient supplementation only take 4.1 days before oviposition begins. Observations have found that at low temperatures, most *S. guani* wasps are inactive and not proactive in searching for and stinging hosts. However, as the temperature increases, their parasitoid behavior changes significantly. This suggests that releasing *S. guani* wasps for pest control when the temperature is above 24 °C can significantly improve their parasitism efficiency.

Within a suitable temperature range, the developmental rate generally accelerates as the temperature rises, but the temperature range for maximum fecundity is often narrower [13,14]. This study found that under five temperature conditions from 21 °C to 33 °C, the developmental rates for the egg, larval, and pupal stages of *S. guani* all increased with rising temperature, with their corresponding developmental durations shortening. At 33 °C, the egg, larval, pupal, and egg-to-adult durations were shorter by 2.9 d, 6.5 d, 30 d, and 38.9 d, respectively, compared to those at 21 °C. However, the optimal temperature range for the reproduction of *S. guani* was only between 27 °C and 30 °C, with both low and high temperatures being unfavorable in this regard. At 21 °C, the total number of offspring was 31.4 individuals/tube, which was 11.6 individuals/tube fewer than the maximum. The reduction of 13.7 offspring at 33 °C represents a 30% decrease in reproductive output, which could significantly affect mass-rearing efficiency.

Under rising temperature conditions, the developmental duration of parasitoids at immature stages does not invariably shorten. For example, the developmental duration of *Venturia canescens* gradually shortens as the temperature increases from 15 °C to 30 °C, but it becomes prolonged when the temperature exceeds 30 °C [26]. Similarly, the developmental duration of *Zele chlorophthalmus* shortens with increasing temperature, but it becomes longer at 29 °C than at 25 °C [27]. In the temperature range studied here, the phenomenon of a prolonged developmental duration with increasing temperature was not observed for *S. guani*. This may be because the set temperatures did not reach the species’ non-optimal developmental threshold; that is, 33 °C is still a suitable developmental temperature for *S. guani*. This result is consistent with a previous study, which found that temperatures needed to rise to 35 °C to be unfavorable for the parasitism and development of *S. sichuanensis* [28].

Parasitoids reared under constant laboratory temperatures for long periods may face greater challenges when confronted with variable temperature conditions in the field. Particularly in the context of current global climate warming, parasitoids are experiencing increased frequency of heat stress. Research indicates that parasitoids have a unique nervous system that can perceive changes in the external environment and adapt by adjusting physiological and biochemical processes [29,30]. When facing heat stress, parasitoids can utilize protective enzymes such as Catalase (CAT), Peroxidase (POD), and Superoxide dismutase (SOD) to maintain the dynamic equilibrium of oxidative metabolism in their bodies [31,32,33]. High temperatures can lead to protein degradation in insects, thereby affecting their cellular functions [34]. Studies show that the genes responsible for thermotolerance in insects are primarily concentrated in the heat shock protein (HSP) family [35]. Generally, the expression level of HSPs in insects is positively correlated with their thermotolerance [36,37]. Research has shown that the expression of the heat shock protein *Tchsp70* in *Trichogramma chilonis Isshii* increased by 7.41-fold and 13.47-fold at 32 °C and 40 °C, respectively, compared to at a control temperature (25 °C) [38]. In our preliminary research, we have already detected heat shock protein genes in wasps of the genus *Sclerodermus*. Our team will subsequently conduct systematic research on their functions to clarify the adaptive mechanisms of *Sclerodermus* in response to heat stress, providing a basis for breeding heat-tolerant strains of parasitoids.

Studies have shown that *Sclerodermus* parasitoids are of a completely inbreeding type; males mate with their female siblings from the same brood immediately after eclosion [39]. This may be associated with the low population density of these parasitoids in the wild and the difficulty in finding non-sibling mates, as they live within the boring tunnels of wood-boring pests. To ensure the presence of male offspring in the subsequent generation, female wasps of the genus *Sclerodermus* will first lay unfertilized eggs, which develop into males. However, an excess of males is detrimental to population development [40]. Therefore, the natural sex ratio of many bethylid wasps is highly stable and female-biased [16,39]. Research indicates that factors such as the number of maternal females, host size, and temperature all affect the offspring sex ratio [41,42]. This study found that temperature has a minor effect on the female sex ratio of *S. guani*; across all five temperatures, the female ratio was greater than 90%, demonstrating the stability of this biological characteristic in *S. guani*.

Parasitoids of the genus *Sclerodermus*, represented by *S. guani*, *S. sichuanensis*, *S. pupariae*, and *S. alternatusi*, are important natural enemy insects widely used in Chinese forestry. Billions of these wasps need to be released annually to control pests [4,43,44]. Inundative release of natural enemies requires the support of robust factory-based mass-rearing technology [45]. An increase in temperature is clearly beneficial in shortening the reproductive cycle of *Sclerodermus* wasps. However, at high temperatures, not only does the parasitism rate of the wasps decrease, but the factitious hosts are also more prone to becoming moldy and turning black, and the number of offspring significantly declines. In summary, we believe that rearing temperatures that are too high or too low are detrimental to the reproductive fitness of *S. guani*. According to the fecundity and parasitism rate of the wasp, the optimal temperature range for the reproduction of *S. guani* is only between 27 °C and 30 °C, and the optimal temperature at which they may be released in the field for pest control is above 24 °C.

## 5. Conclusions

Temperature significantly affects the parasitism capacity and behavior of *Sclerodermus guani*. At low (21 °C) and high (33 °C) temperatures, the parasitism rate is lower than at 24–30 °C. The higher the temperature, the more actively the female wasp seeks out and stings the host, and the shorter its pre-oviposition period.

The developmental duration of *S. guani* shortens as temperature increases. Both high (33 °C) and low (21 °C) temperatures are detrimental to the reproduction of *S. guani*. The observed reduction of 13.7 offspring at 33 °C represents a 30% decrease in reproductive output.

When temperatures exceed 30 °C, although the parasitism rate remains above 80%, the number of eggs laid per female decreases to 60% of that at a normal temperature. This will directly affect the economic efficiency of mass rearing and the parasitism effectiveness in the field. We recommend prioritizing the release of wasps during the 27–30 °C period in high-temperature regions, along with implementing short-term cold storage and transportation protocols.

## Figures and Tables

**Table 1:** Parasitism rate and emergence rate of *Sclerodermus guani* reared at different temperatures.

Temperature/°C	Parasitized Host/n	Parasitism Rate/%	Number of Hosts with Parasitoid Offspring/n	Emergence Rate/%
21	20	66.67 b	14	46.67 b
24	29	96.67 a	29	96.67 a
27	27	90.00 ab	27	90.00 a
30	26	86.67 ab	26	86.67 a
33	25	83.33 ab	25	83.33 a
*χ* ^2^	/	11.606	/	29.667
*p*	/	0.021	/	<0.001

Note: Data are means ± SE of thirty replicates. Values with different lowercase letters in the same column are significantly different at the 0.05 level according to Fisher’s exact test.

**Table 2:** The developmental rates of *Sclerodermus guani* among different groups.

Temperature (°C)	Developmental Rates		
	Egg Stage	Larval Stage	Pupal Stage
21	0.1368	0.0932	0.0244
24	0.2034	0.1365	0.0653
27	0.2506	0.1626	0.0684
30	0.2807	0.1678	0.0758
33	0.2867	0.1741	0.0908

**Table 3:** The lower developmental threshold temperature and effective accumulated temperature of *Sclerodermus guani*.

Stage	Lower Developmental Threshold Temperature (°C)	Effective Accumulated Temperature (Degree-Days)	Model	*R* ^2^	*F*	*p*
Egg stage	10.19	72.57	*y* = 72.57*x* + 10.19	0.912	31.148	0.011
Larval stage	7.73	131.21	*y* = 131.21*x* + 7.73	0.845	16.302	0.027
Pupal stage	15.57	176.02	*y* = 176.02*x* + 15.57	0.841	15.846	0.028

## Data Availability

The original contributions presented in this study are included in the article/Supplementary Materials. Further inquiries can be directed to the corresponding authors.

## Supplementary Materials

The following supporting information can be downloaded at: https://www.mdpi.com/article/10.3390/biology14091234/s1, Table S1: Difference in Duration (days) is a record of the duration of each stage; Table S2: Offspring Count shows the number of male and female offsprings and the sex ratio; Table S3: Female Sex Ratio shows the Female Sex Ratio and Female Sex Ratio after Natural Logarithm transformation.

## Abbreviations

The following abbreviations are used in this manuscript:

RH	relative humidity
ANOVA	analysis of variance
LSD	least significant difference
SE	standard error
CAT	catalase
POD	peroxidase
SOD	superoxide dismutase
HSPs	heat shock proteins
